# Pre-Hospital Glyceryl Trinitrate: Potential for Use in Intracerebral Hemorrhage

**DOI:** 10.4172/2329-6895.1000141

**Published:** 2013-11-14

**Authors:** Jason J Chang, Nerses Sanossian

**Affiliations:** 1 Department of Neurology, University of Southern California, USA; 2 Roxanna Todd Hodges Comprehensive Stroke Clinic, University of Southern California, USA

**Keywords:** Intracerebral hemorrhage, Antihypertensive, Glyceryl trinitrate, Nitroglycerin, Treatment, Blood pressure

## Abstract

**Background:**

Intracerebral hemorrhage is associated with poor clinical outcome and high mortality. Research and treatment modalities have focused on the expansion of the primary hematoma through blood pressure control and activation of coagulation factors. However, clinical trials have failed to show decreased rates of death or disability in intracerebral hemorrhage following hospital initiation of blood pressure control. However, as clinical deterioration often occurs immediately after onset, pre-hospital initiation of blood pressure control may be more ideal.

**Methods:**

Relevant terms in the National Library of Medicine PubMed database and selected research including basic science, translational reports, meta-analyses, and clinical studies were searched.

**Results:**

Trends indicating improved clinical outcome in intracerebral hemorrhage after hospital-initiated intensive systolic blood pressure control (goal<140 mmHg) have been demonstrated. Statistical significance may not have been obtained because of late treatment times of blood pressure control that approached median 4–6 hours after clinical onset. One trial utilizing glyceryl trinitrate in the pre-hospital setting has been shown to significantly decrease blood pressure within fifteen minutes and improve 90-day clinical outcome.

**Conclusions:**

Glyceryl trinitrate represents an ideal pre-hospital blood pressure medication because it can be delivered via sublingual or transdermal routes, has a quick and graded onset of action, has neuroprotective effects, maintains cerebral perfusion, and has an established record of safety. As intracerebral hemorrhage requires prompt action to prevent clinical deterioration, more emphasis on pre-hospital therapies for blood pressure reduction will become essential in future therapies.

## Introduction

Of an estimated 795,000 new strokes per year, intracerebral hemorrhage (ICH) accounts for 10% of all strokes [1]. Mortality associated with ICH continues to be significantly worse than in ischemic stroke. In the last 15 years, the one-month mortality rate after ICH has remained unchanged at 44% [2], one-year survival rate remains 38% [3], and Kaplan-Meier analysis shows an abysmal 16-year cumulative survival of only 3.2–9.8% [4].

The primary phase of ICH–hematoma expansion–remains the target of research and treatment as rapid expansion has been associated with neurological deterioration and worse outcome [5,6]. Although several factors including larger initial hematoma and heterogeneous density have been associated with greater ICH expansion [7], treatment options in this primary phase of ICH have focused on preventing hematoma expansion through blood pressure reduction in hypertensive patients and activation of coagulation.

While promoting coagulation has the potential for reducing expansion, it has not been shown to improve outcome or reduce disability [8]. However, Factor VII therapy will continue to be evaluated as identification of the CT-angiography spot sign has allowed for greater selectivity in predicting ICH expansion [9,10]. On the other hand, early aggressive blood pressure management within the hospital has been shown to decrease hematoma growth with the potential of reducing disability at ninety days [11,12].

## Pre-Hospital Clinical Deterioration in Intracerebral Hemorrhage

Many patients presenting to the emergency department with ICH will have already deteriorated due to hemorrhage expansion. Studies using CT imaging report hematoma growth in greater than 70% of patients within the first three hours of symptom onset [13] with only 11–12% expanding after the first three hours [14]. Furthermore, prehospital studies showed that substantial neurological deterioration occurred in 22% of patients with ICH between the time of paramedic arrival and emergency department admission. Clinically, as many as three in ten patients who are initially alert during paramedic evaluation within the first two hours of onset will have significantly deteriorated before arrival to the hospital [15]. These patients with pre-hospital neurological deterioration had mortality rates of 77%. Initial diastolic blood pressure (DBP) also correlated with clinical deterioration [16].

These studies show that the vast majority of hematoma expansion occurs during the initial phase. This is the period of time when initial symptoms are recognized and where hematoma expansion is most volatile as evidenced by the CTA spot sign. Treatment outside of this phase is unlikely to yield significant effect on outcome, and the earliest time windows for treatment, including pre-hospital treatment of blood pressure may be needed to prevent clinical deterioration and obtain optimum clinical results.

## Blood Pressure Control as a Therapeutic Target in Intracerebral Hemorrhage Outcome

CT-perfusion studies have shown that intensive blood pressure control does not precipitate perihematomal ischemia in ICH patients [17]. As intensive blood pressure control is safe and early hematoma growth is associated with increased mortality, prevention of this expansion through intensive blood pressure control represents a logical therapeutic target in ICH. Two major trials address the role of early intensive hospital-based blood pressure control.

In the Intensive Blood Pressure Reduction in Acute Cerebral Hemorrhage Trial (INTERACT) pilot, intensive systolic blood pressure (SBP) control (goal<140 mmHg) resulted in significantly less mean proportional hematoma growth (P=0.04) and relative risk of hematoma growth (P=0.05) [11]. These promising results lead to INTERACT2, which randomized 2794 patients to intensive SBP control (goal<140 mmHg) and guideline recommended SBP goals (goal<180 mmHg). The primary outcome of death or disability was not significantly decreased (P=0.06). However, the intensive blood pressure group significantly favored secondary outcome measures such as ordinal analysis of modified Rankin scores with favorable shift of Rankin score distributions (P=0.04) and physical and psychological well-being as manifested by anxiety and depression (P=0.05), pain or discomfort (P=0.01), and self-care (P=0.02) [18].

In the Antihypertensive Treatment of Acute Cerebral Hemorrhage (ATACH) pilot trial, 60 patients were enrolled in three tiers of increasingly intensive blood pressure goals. Primary clinical outcomes (death and severe disability defined by Rankin scores of 4 to 6) were measured at 90 days. Although primary outcome did not meet statistical significance, measured variables such as hematoma expansion, perihematomal edema, and 90-day Rankin score all showed trends favoring the intensive blood pressure control group [19]. As the pilot trial was underpowered, ATACH II, a multi-center randomized phase three trial, is currently recruiting patients to evaluate the hypothesis that intensive blood pressure lowering (goal SBP<140 mmHg) will lead to reduced death and disability outcomes at 90 days [20].

Several reasons may explain the lack of statistical significance for primary outcome found in INTERACT2 and the ATACH pilot. First may be a greater relevance of secondary mechanisms of injury in ICH–perihematomal edema, blood-brain-barrier destruction, and upregulation of inflammatory mediators–towards overall clinical outcome [21]. Second may be the relatively late times initiating therapy in both the ATACH pilot trial and INTERACT2. In ATACH, the treatment time was mean six hours after onset. In INTERACT2, median treatment time in the intensive group and standard group were 4 and 4.5 hours, respectively. However, subgroup analysis of the INTERACT pilot by quartile time periods demonstrated significant reductions in proportional hematoma growth (measured at 72 hours) ranging from 22% reduction for the <2.9 hour treated group to 3% reduction for the > 4.9 hour treated group (P=0.001). Absolute hematoma growth, while also showing a similar trend favoring earlier treated groups, did not meet statistical significance (P=0.12) [22]. These findings suggest that earlier treatment of blood pressure may result in decreased hematoma volume expansion and improvement in clinical outcome. One may conclude that blood pressure control in ICH should be started as early as possible in the pre-hospital setting.

## Glyceryl Trinitrate as a Promising Medication for Stroke Treatment

Glyceryl trinitrate (GTN) as a pre-hospital therapy in ICH is promising because of four properties: (1) alternative entry routes besides oral or intravenous (many ICH patients will be obtunded or have facial and swallowing weakness rendering delivery by mouth unsafe), (2) quick and graded onset of action, (3) neuroprotective effects, and (4) established record of safety.

Transdermal and sublingual GTN have been studied for many years in myocardial ischemia, congestive heart failure, and ischemic stroke. GTN acts primarily via vascular smooth muscle relaxation, which leads to arterial and venous vasodilation. This has the effect of decreasing cardiac output and improving myocardial oxygen supply-to-demand ratio. The rapid metabolism of oral GTN is circumvented through transdermal and sublingual routes. With a more rapid onset time (3 minutes) and shorter duration (10–30 minutes), sublingual GTN can be used in combination with slower onset (30–60 minutes) and longer duration transdermal GTN [23]. Transdermal and sublingual GTN showed similar mean reductions in systolic blood pressure of 9.80 mmHg [24] and 14 mmHg [25], respectively. Studies of transdermal GTN demonstrated that it could lower peripheral blood pressure, central blood pressure, and pulse pressure without reducing middle cerebral artery velocities and without affecting platelets [26–29].

GTN has also demonstrated neuroprotective properties in preclinical stroke models. GTN acts as an inhibitor of apoptosis [30] through formation of an NO+ equivalent molecule that nitrosylates the redox modulatory site at the NMDA receptor. This has the neuroprotective effect of inhibiting NMDA receptor-mediated neurotoxicity [31]. In cerebral ischemia-reperfusion models, administration of NO donor was shown to decrease free radical levels [32] and reduce brain infarct volume [33], likely through release of NO, which increases blood flow to collateral blood vessels [29] and reduces monocyte and neutrophil adhesion and migration [34].

Extensive safety experience with transdermal GTN in both ischemic stroke and ICH is currently being evaluated in the Efficacy of Nitric Oxide in Acute Stroke (ENOS) trial. ENOS is enrolling 3500 acute ischemic stroke and ICH patients randomizing to GTN patch (5 mg over 24 hours) or placebo. Trial enrollment is anticipated to complete in October 2013 and is nearing completion with final results anticipated within the year. Initial results from ENOS have already provided strong reassurance regarding the safety of GTN with the evaluation committee recommending completion of the trial. Included in GTN’s excellent safety profile is its ability to lower blood pressure without decreasing cerebral perfusion as evidenced by preliminary data publications from the ENOS group that did not show worse 90-day clinical outcome in patients with significant (>50%) carotid stenosis [35]. Additionally, transcranial Doppler, SPECT, and xenon CT studies of cerebral blood flow have shown that transdermal GTN increases or maintains cerebral perfusion in acute ischemic stroke patients despite decreases in mean arterial pressure [29,36,37].

In ICH, theoretical concerns regarding venodilatory effects on intracranial pressure (ICP) are nullified by actual physiologic studies in humans. In individuals with normal starting ICPs, GTN produces only minimal and transient increases in ICP [38]. In individuals with acute hemorrhagic intracranial lesions, GTN most commonly produces no elevation or even a mild reduction in ICP, likely because the raised ICP overrides the weak venodilatory signal [29,38–40].

Recently, a single-center, single-blinded randomized controlled study, the Rapid Intervention with Glyceryl Trinitrate in Hypertensive Stroke Trial (RIGHT) evaluated the use of pre-hospital transdermal GTN in patients with ischemic stroke and ICH. Paramedics randomized 41 patients (25 in the GTN group and 16 placebo). The GTN group had SBPs significantly reduced by 21 mmHg at 15 minutes (P=0.049) and 18 mmHg at 2 hours (P=0.030). Blood pressure reduction was associated with improved functional outcome and significantly better 90-day Rankin score in the GTN group (P=0.017). Post-hoc analysis also showed a promising relationship with a correlation coefficient of −0.296 (P=0.06) [41].

## Alternative Drugs for Pre-hospital Blood Pressure Control

**Figure 1:**
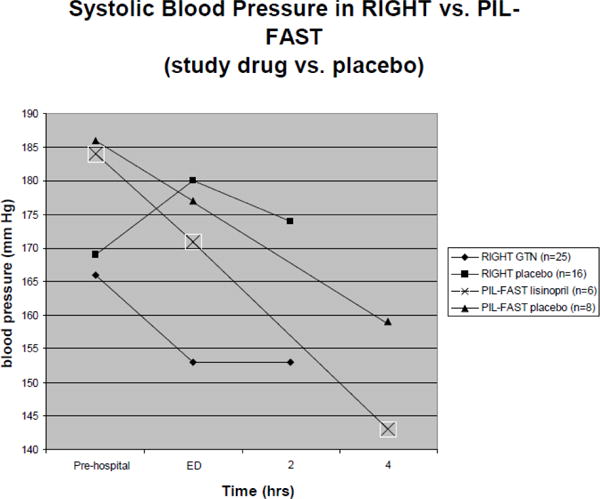
Comparison of Systolic Blood pressures in Pre-hospital Administration of Glyceryl Trinitrate (RIGHT trial) and Lisinopril (PIL-FAST trial). **Systolic blood pressures in RIGHT were measured at pre-hospital, Emergency Department (ED) admission, and hour 2. Systolic blood pressures in PIL-FAST were measured at pre-hospital, ED admission, hour 4, hour 24 (not shown), and day 7 (not shown).

Currently only two blood pressure medications have been studied for pre-hospital blood pressure control: GTN studied in RIGHT and lisinopril studied in PIL-FAST. In PIL-FAST, paramedics administered a crushed 5 mg tablet of lisinopril sublingually. This medication was continued over 7 days. As a small pilot trial, 6 patients received lisinopril compared to 8 patients in the placebo arm. Comparison of pre-hospital lisinopril and GTN administration on systolic blood pressures is shown in Figure 1. As a small pilot trial, statistically significant differences in blood pressure reduction were not found between the lisinopril and placebo group. In addition, adverse events–including increase in serum creatinine, chest infection, and hypotension–were noted in the lisinopril group. Only 4 of the 14 patients completed their medication course [42].

## Pre-hospital Differentiation of ICH from Ischemic Stroke

Ideally, patients with diagnosed ICH would be recruited into prehospital clinical trials of therapy. Realistically however, stroke subtype cannot be diagnosed without imaging as clinical deficits in ICH and ischemic stroke are based on lesion sites that can occur in either disease. Preliminary data on 538 patients consecutively enrolled in FAST-MAG (with 134 patients having ICH) did show ICH associated with higher SBP and DBP, younger age, less frequent atrial fibrillation, higher median Los Angeles Motor Scores (LAMS), male gender, Hispanic ethnicity, and earlier evaluation times in relation to ischemic stroke [43].

A more recent multivariable analysis of a larger dataset of 731 cases (164 with ICH) was performed by randomly assigning to derivation and validation datasets, using all variables available at time of paramedic encounter in a backward stepdown linear, additive logistic regression and classification tree analysis. The performance of a simple logistic model using seven binary predictors (age ≤ 56yo, non-black ethnicity, SBP>147, DBP>103, Los Angeles Motor Scores>3, no atrial fibrillation, no previous stroke) showed a sensitivity of 87.2% and specificity of 67.3% with overall accuracy of 77.2%. The validation dataset showed 75.4% specificity and 72.4% sensitivity [44]. These background studies suggested that a pre-hospital administered scale based on clinical variables could achieve modest accuracy in differentiating ICH from ischemic stroke, but is not effective enough in patient recruitment.

## Safety and Feasibility of pre-Hospital Anti-Hypertensive Therapy in Ischemic Stroke

Pre-hospital use of anti-hypertensive treatment for ICH patients not verified by imaging will inevitably treat ischemic stroke patients. The International Stroke Trial (IST) found a U-shaped distribution of blood pressure outcomes, with both higher and lower pressures associated with poor outcomes [45]. However over the past decade, evidence has suggested that early lowering of blood pressure in patients with ischemic stroke and severe hypertension is safe and possibly beneficial. In ischemic stroke, large-scale observational studies have consistently shown that elevated blood pressure is associated with worse outcomes [46–48]. In fact, elevated blood pressure in the setting of failed autoregulation may increase cerebral edema and hemorrhagic transformation [49]. Several randomized clinical trials, including ACCESS, COSSACS, and CHHIPS, have now reported that early initiation of antihypertensive therapy in acute ischemic stroke is safe and efficacious and may improve clinical outcome [50–52].

Moreover, for patients eligible for thrombolysis who are also severely hypertensive with SBP>185 mmHg, early initiation of antihypertensive therapy to reach this goal SBP is supported by positive results in multiple clinical trials [53]. In fact, active initiation of early blood pressure lowering is a recommended practice in the United States and international treatment guidelines. Pre-hospital initiation of blood pressure control is safe and can shorten the time required for the patient to be eligible for thrombolysis.

## Conclusion

Intracerebral hemorrhage is associated with poor clinical outcome and high mortality. Efforts to improve mortality have largely focused on hematoma expansion through intensive blood pressure control. Hospital-based blood pressure control may often be too late as clinical deterioration can occur immediately after ICH onset. To date, only lisinopril and GTN have been evaluated as pre-hospital medication for blood pressure control. GTN represents an ideal pre-hospital blood pressure control medication because it can be delivered via alternative transdermal or sublingual routes, has a quick and graded onset of action, has neuroprotective effects, and has an established record of safety. Its feasibility and effectiveness as a pre-hospital blood pressure medication has been evaluated through RIGHT, which showed a significant early reduction of blood pressure. As acute neurological injury requires prompt action, more emphasis on pre-hospital therapies such as GTN will become essential in future therapies (Table 1).

## Figures and Tables

**Table 1:** Summary of important trials regarding Blood Pressure Control, ICH expansion, and Glyceryl Trinitrate therapy in Intracerebral Hemorrhage.

Trial	Study type	Sample size	Conclusion	Limitations
**INTERACT2-Anderson et al. **[18]	Multi-center, prospective, randomized, blinded	2794	-Ordinal analysis showed significantly lower 90-day Rankin scores (P=0.04) and psychological well-being in intensive blood pressure	-no standard blood pressure lowering medication used -intensive blood pressure lowering did not result in reduction of primary outcome (death or major disability)
**ATACH pilot-Qureshi and Palesch **[20]	3-tier, single-center, prospective, blinded	60	-no significant relationship between degree of SBP reduction and hematoma volume, perihematomal edema, and 90-day Rankin score	-underpowered to prove significant relationships between SBP and outcome variables -different time points for initial recording of SBP and hematoma volume, making it impossible to determine if expansion simply occurred because of earlier initial recording
**PREDICT-Demchuk et al.** [9]	Multi-center, prospective, observational, cohort	268	-CTA spot sign resulted in significantly higher 90-day mortality (P=0.001), higher 90-day Rankin score (P<0.001), absolute ICH growth (P<0.001) -sensitivity of CTA spot sign for hematoma expansion=51%, specificity=85%	-less robust pos-predictive and neg-predictive value with large variability -CTA spot sign reliability required neuroradiologist with less robust reliability for untrained site investigator -variability in timing of contrast bolus among institutions; increased yield may result from second-pass imaging completed 1 min after contrast bolus
**RIGHT-Ankolekar et al.** [41]	Single-center, prospective, single-blinded, randomized control	41	-prehospital GTN significantly lowered SBP at 2 hrs (P=0.030) and improved 90-day Rankin score (P=0.017)	-small sample size -single center
**ENOS-Bath et al.** [27]	Multi-center, prospective, blinded, randomized control	3500 (estimated)	-safety and efficacy of transdermal GTN in 90-day clinical outcome	-n/a

-SBP=systolic blood pressure

-GTN=glyceryl trinitrate
